# Healthy Diets and Modifiable Risk Factors for Non-Communicable Diseases—The European Perspective

**DOI:** 10.3390/foods9070940

**Published:** 2020-07-16

**Authors:** Marcello Iriti, Elena Maria Varoni, Sara Vitalini

**Affiliations:** 1 Department of Agricultural and Environmental Sciences, Milan State University, Via G. Celoria 2, 20133 Milan, Italy; sara.vitalini@unimi.it; 2 Department of Biomedical, Surgical and Dental Sciences, Milan State University, Via Beldiletto 1/3, 20122 Milan, Italy; elena.varoni@unimi.it

**Keywords:** Mediterranean diet, Nordic diet, overweight, obesity, cardiovascular disease, functional foods, nutraceuticals, bioactive phytochemicals

## Abstract

Non-communicable diseases pose a serious threat to Western countries, in particular to European populations. In this context, healthy diets, such as the Mediterranean diet and the New Nordic diet developed in 2004, in addition to other healthy lifestyle choices (i.e., regular and low to moderate intensity levels of physical activity) can contribute to reduce the risk factors associated with cardiovascular disease and type 2 diabetes (majorly preventable, diet-related, non-communicable diseases), including being overweight, obesity, hypertension, hyperglycemia and hypercholesterolemia. The Mediterranean diet and the Nordic diet share common traits: they are rich in nutrient-dense foods (mostly plant-derived foods) and low in energy-dense foods (mainly of animal origin). However, more studies are needed to ascertain the long-term effects of adherence to both dietary styles with regards to disease prevalence and incidence, especially for the New Nordic Diet.

In the few last decades, the benefits of dietary styles rich in nutrient-dense foods have been emphasized in terms of longevity, healthy ageing and morbidity. Indeed, diets including plenty of plant foods have been associated with a reduced risk and incidence of chronic degenerative diseases such as cardiovascular disease, type 2 diabetes, metabolic syndrome, neurodegenerative disorders and some cancers. In general, healthy dietary habits include a low consumption of refined sugars, salt, saturated and trans fats, as well as high intake of fruit, vegetables (including legumes, whole grain cereals and nuts), low-fat dairy products and healthy lipids (from plant oils and seafood).

According to the World Health Organization (WHO), non-communicable diseases, including cardiovascular disease, cancers, respiratory diseases and diabetes, are responsible for almost 70% of all deaths worldwide. The rapid rise of non-communicable diseases has been driven by a number of (modifiable) behavioral risk factors, such as unhealthy diets, physical inactivity, exposure to tobacco smoke and the harmful use of alcoholic beverages, in addition to environmental (air pollutants), occupational (carcinogens, particulates, gases, fumes) and metabolic (overweight/obesity, hypertension, hyperglycemia, hypercholesterolemia) risk factors [1]. Of the six WHO regions, the WHO European Region is the most severely affected by non-communicable diseases (Table 1). Therefore, The European Food and Nutrition Action Plan 2015–2020 aimed at significantly reducing the burden of preventable diet-related non-communicable diseases, obesity and all other forms of malnutrition prevalent in the region, with emphasis on the decrease in the prevalence of obesity and diabetes, as well as overweight children under five years old [1].

A number of studies have established the health-promoting effects of two European diets: the Mediterranean diet and the Nordic diet, particularly against cardiovascular disease and type 2 diabetes [2]. As substantiated by many observational studies, the Mediterranean diet, low in energy-dense foods, can be considered the archetype of a health-promoting lifestyle by virtue of the phytochemical diversity of its food components (Table 2).

The traditional Mediterranean diet originated in the olive- and grapevine-growing areas of the Mediterranean region and has a strong cultural association with these areas. It is characterized by a high intake of plant-based foods (cereals, fruit, vegetables, legumes and nuts) and olive oil; a moderate intake of fish and poultry; a low to moderate intake of red wine; and a low intake of dairy products (principally yogurt and cheese), red meat, processed meats and sweets (to which fresh fruit is often substituted). Social and cultural factors closely associated with the traditional Mediterranean diet, including shared eating practices, post-meal siestas (afternoon naps) and lengthy meal times, are also thought to contribute to the attributed positive health effects recorded in the Mediterranean region. However, the Mediterranean diet varies by country and region, despite the common traits, due to the climatic, cultural and religious differences among southern European, northern African and eastern Mediterranean populations [4].

The New Nordic Diet was developed in 2004 by scientists, nutritionists and chefs to address the growing overweight population and obesity rates, as well as the unsustainable farming systems in the Nordic countries (Table 3).

This dietary style shares many characteristics with the Mediterranean diet, but comprises traditional foods from Denmark, Finland, Iceland, Norway and Sweden (please visit the Baltic Sea Diet Pyramid created by the Finnish Heart Association, the Finnish Diabetes Association and the University of Eastern Finland at www.helsinkitime.fi). Staple components of the New Nordic Diet include whole grain cereals (barley, oats and rye), vegetables (cabbage, tubers and root vegetables), legumes (mainly beans and peas), berries and fruit, nuts and seeds, and fish (herring, mackerel and salmon). A notable point of difference is the use of rapeseed (canola) oil instead of olive oil, rich in α-linolenic acid (a type of omega-3 polyunsaturated fatty acid). The Nordic diet is also characterized by a moderate consumption of dairy products and eggs, as well as a low intake of processed foods, sweets (including added sugars and sweetened beverages) and red meat. Not least, the Nordic diet is predominantly plant-based and locally sourced, thus ensuring a more environmentally friendly production with reduced waste when consumed within the Nordic region [2].

The health benefits of the Nordic diet have also been investigated—though to a lesser extent than those of the Mediterranean diet—and associated with improvements in risk factors for both cardiovascular disease and type 2 diabetes. In hypercholesterolemic individuals, the Nordic diet improved their blood lipid profile and insulin sensitivity, in addition to reducing blood pressure [6]. In subjects with metabolic syndrome, the Nordic diet ameliorated the blood lipid profile with beneficial effects on low-grade inflammations [7], besides decreasing the ambulatory blood pressure [8]. These findings, based on randomized clinical trials, were partially confirmed in population-based studies and the association between the adherence to the Nordic diet and cardiometabolic risk factors is still equivocal [9]. Adherence to the Nordic diet was also inversely associated with the risk of type 2 diabetes [10] and also induced weight loss in centrally obese men and women [11]. However, despite the fact that studies have shown that Nordic diet has beneficial effects on the risk factors for diabetes, such as obesity and low-grade inflammation, evidence on the long-term impact of adherence to the Nordic diet on diabetes prevalence and incidence requires larger prospective studies [12].

In conclusion, we have to take into account that the adherence to Mediterranean and Nordic diet may not always be high in the southern and northern European populations, respectively. In other words, the prevalence and mortality rate of cardiovascular disease and diabetes can be high in some of these countries (Figure 1 and Figure 2). Therefore, to assess the adherence to both diets is pivotal in order to evaluate their predictive ability on specific risk factors and biomarkers, by powerful tools such as the Mediterranean diet score [13] and the Baltic Sea diet score [14].

## Figures and Tables

**Figure 1:**
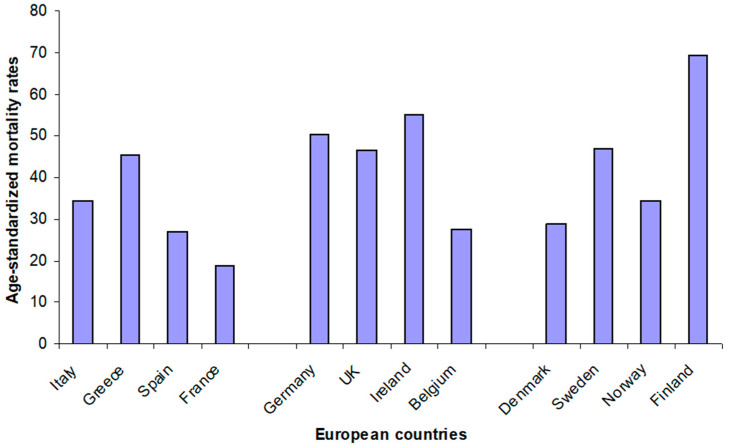
Age-standardized mortality rates per 100,000 population in 2014—ischemic heart diseases (both sexes) in selected European countries (from: WHO Mortality Database, https://apps.who.int/healthinfo/statistics/mortality/whodpms/).

**Figure 2:**
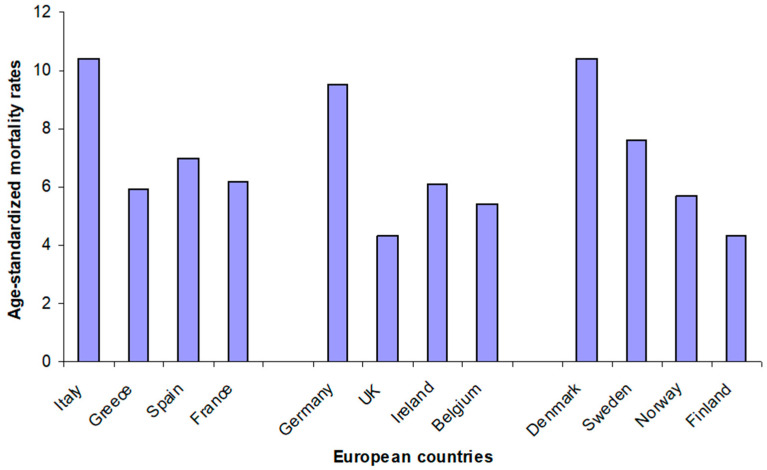
Age-standardized mortality rates per 100,000 population—diabetes mellitus (both sexes) in selected European countries (from: WHO Mortality Database, https://apps.who.int/healthinfo/statistics/mortality/whodpms/).

**Table 1:** Burden of non-communicable diseases, overweight and obesity in the WHO European Region: factsheet.

● Cardiovascular disease, diabetes, cancer and respiratory diseases (the four major NCDs) together account for 77% of the burden of disease and almost 86% of premature mortality
● In 46 countries (accounting for 87% of the Region), more than 50% of adults (aged ≥ 20 years, both sexes) are overweight or obese, and in several countries the rate is close to 70% of the adult population
● Overweight and obesity are estimated to result in the death of approximately 320,000 men and women in 20 western European countries every year
● Rates of overweight and obesity in some parts of eastern Europe have risen more than threefold since 1980
● Overweight and obesity are also highly prevalent among children and adolescents, particularly in southern European countries
● The prevalence of overweight and obesity was 11–33% for children aged 11 years, 12–27% for children aged 13 years and 10–23% for those aged 15 years

Adapted from [1].

**Table 2:** Dietary pattern and lifestyles of traditional Mediterranean diet.

Dietary Habits
1. High consumption of minimally-processed, local and seasonal plant food (whole-grain cereals, fresh fruit, cooked and raw vegetables, nuts)
2. Daily fat intake ranging from 25% to 35% of energy (with saturated fat ranging from ≤7% to 8% of energy)
3. Daily intake of low to moderate amounts of dairy products (mainly low-fat cheeses and yogurt)
4. Twice-weekly consumption of low to moderate amounts of fish and poultry; up to seven eggs per week
5. Fresh fruit as the typical dessert, with sweets containing sugars or honey consumed only a few times per week
6. Consumption of red meat only a few times per month
7. Regular, low to moderate consumption of wine at main meals; approximately 1-2 glasses per day for men and 1 glass for women (optional)
8. Herbs and spices to season food rather than salt or fat
**Lifestyles**
1. Regular daily physical activity
2. Enjoy meals with others (family and friends)

Adapted from [3].

**Table 3:** Guidelines of the New Nordic Diet.

1. Eat more fruit and vegetables every day
2. Eat more whole grain products
3. Eat more food from the sea and lakes
4. Eat higher-quality meat, and less of it
5. Eat more food from wild landscapes
6. Eat organic products whenever possible
7. Avoid food additives
8. Eat more meals based on seasonal products
9. Eat more home-cooked food
10. Produce less waste

Adapted from [5].
